# Posterior C1-C2 fixation for atlantoaxial instability due to a dysplastic C1 arch, complicated by an accidental vertebral artery injury managed with intravascular intervention and salvage contralateral unilateral fusion

**DOI:** 10.1093/jscr/rjaf267

**Published:** 2025-05-05

**Authors:** Armand Dominik Škapin, Peter Brumat, Juš Kšela, Miha Vodičar

**Affiliations:** Faculty of Medicine, University of Ljubljana, Vrazov trg 2, Ljubljana 1000, Slovenia; Department of Orthopaedic Surgery, University Medical Centre Ljubljana, Zaloška 9, Ljubljana 1000, Slovenia; Faculty of Medicine, University of Ljubljana, Vrazov trg 2, Ljubljana 1000, Slovenia; Department of Orthopaedic Surgery, University Medical Centre Ljubljana, Zaloška 9, Ljubljana 1000, Slovenia; Faculty of Medicine, University of Ljubljana, Vrazov trg 2, Ljubljana 1000, Slovenia; Department of Cardiovascular Surgery, University Medical Centre Ljubljana, Zaloška 7, Ljubljana 1000, Slovenia; Faculty of Medicine, University of Ljubljana, Vrazov trg 2, Ljubljana 1000, Slovenia; Department of Orthopaedic Surgery, University Medical Centre Ljubljana, Zaloška 9, Ljubljana 1000, Slovenia

**Keywords:** congenital, dysplasia, atlantoaxial instability, C1-C2, vertebral artery injury, unilateral fusion

## Abstract

Posterior fixation with fusion is recommended in symptomatic congenital anomalies of the upper cervical spine. Altered vertebral anatomy in such cases increases the likelihood of intraoperative complications, where injury to the vertebral artery (VA) may result in cerebellar and dorsolateral medulla oblongata ischemia. Prompt and appropriate intervention is paramount if complications arise. We present a patient with a dysplastic C1 arch, leading to atlantodental arthrosis and anterior C1 subluxation with resultant compressive myelopathy of the spinal cord. During elective surgery, a VA injury occurred, which was managed with intravascular intervention and salvage contralateral posterior unilateral C1-C2 fusion. Postoperatively, the patient developed partial Wallenberg syndrome. Imaging revealed two ischemic changes in the cerebellar hemisphere. One year after surgery, the patient showed substantial improvement following comprehensive rehabilitation, able to ambulate without support and maintain a significant level of independence in everyday activities.

## Introduction

Congenital anomalies of the cervical vertebrae vary widely, from simple, asymptomatic conditions to complex malformations with severe structural and neurological complications [1, 2]. Structural abnormalities and instability can accelerate degenerative processes, causing initially asymptomatic conditions to become symptomatic over time and potentially lead to neurological compression [2]. When accompanied by spinal cord myelopathy with radiographic confirmation, surgical decompression is indicated, with or without fusion [2]. The procedure aims to relieve pressure on neurological structures and is generally safe. However, altered vertebral and vascular anatomy increases the risk of complications, including damage to adjacent vital structures, making prompt and appropriate intervention crucial [3].

We present a case of a 46-year-old female with a dysplastic C1 arch, resulting in atlantodental arthrosis with anterior C1 subluxation and compressive myelopathy. During elective surgery, a vertebral artery (VA) injury occurred, which was managed with intravascular intervention and salvage posterior contralateral unilateral C1-C2 fusion.

## Case report

**Figure 1:**
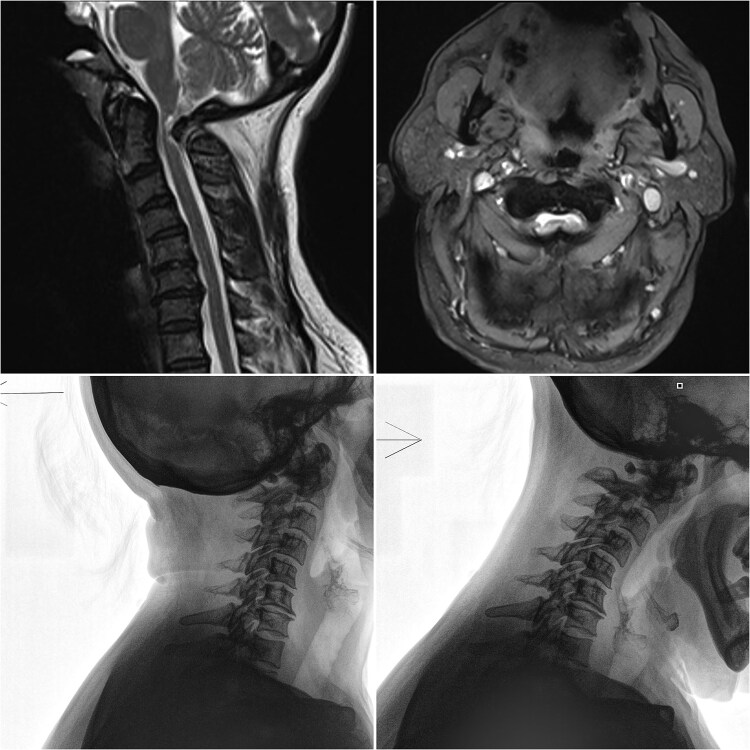
Sagittal and axial MRI, along with flexion/extension X-rays of the cervical spine, demonstrating anterior C1 subluxation, central canal narrowing, and myelopathy.

A 46-year-old female presented with gait instability and decreased sensation in her limbs. Clinical examination revealed an ataxic gait, hyperreflexia, and left-sided hemispasticity. Magnetic resonance imaging (MRI) of the cervical spine showed C1-C2 arthrosis and anterior C1 subluxation (Fig. 1), causing central canal narrowing and myelopathy. Surgical treatment was planned, including C1 laminectomy, and C1-C2 posterior fixation with fusion. A preoperative CT vertebral angiography was performed.

The surgical procedure began with a posterior midline approach to the occiput, C1, C2, and C3. Screws were successfully placed in the right C1 lateral mass and C2 pedicle, but an attempted screw insertion into the left C1 lateral mass caused a breach due to dysplastic bony anatomy, resulting in significant bleeding from a suspected VA injury upon removing the drill, initially managed with tamponade. A cardiovascular surgeon assessed the injury and determined that direct suturing was not feasible due to the VA's anatomical position, recommending intravascular intervention. After consulting with an interventional neuroradiologist, endovascular management was chosen.

**Figure 2:**
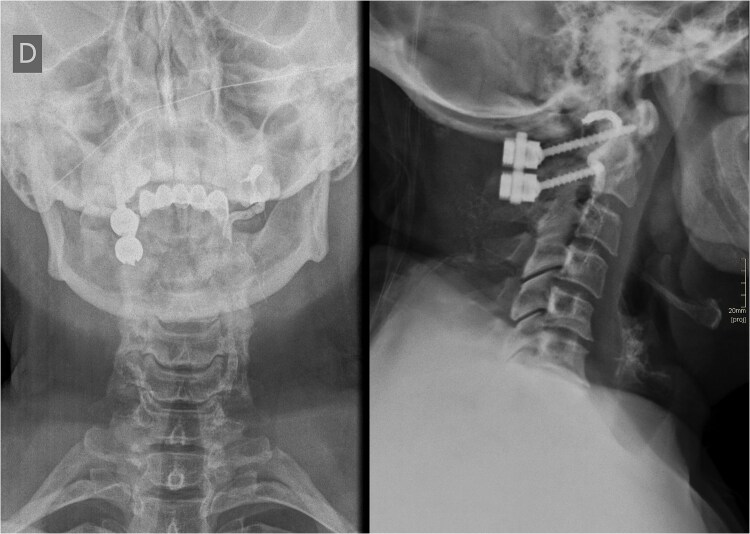
Postoperative X-ray of the cervical spine showing contralateral unilateral C1-C2 fixation and the left VA opacified with a radiopaque contrast agent.

Before transferring the patient to the interventional radiology suite, a salvage contralateral unilateral C1-C2 fixation with C1 laminectomy and posterolateral fusion was performed, achieving full spinal cord and dura decompression. CT angiography revealed complete left VA occlusion at the C1 groove, likely from thrombosis, with the right VA patent and dominant. To prevent rebleeding during revascularization, the injured VA was occluded using coils and liquid embolic agents (Fig. 2).

A head CT and CT angiography of the cervical and cerebral arteries, performed on the same day after surgery, revealed no intracranial hemorrhage or other pathological changes, along with the embolized segment of the left VA. All other arteries, including the left posterior inferior cerebellar artery (PICA), appeared patent. A brain MRI conducted the following day showed two ischemic lesions in the left cerebellar hemisphere and hyperintense signal in the dorsal spinal cord at C1-C2, suggestive of post-decompression edema. The patient was started on aspirin, prophylactic anticoagulation, and corticosteroids.

In the subsequent days, she developed partial Wallenberg syndrome, with dysarthria, left-sided miosis, facial sensory loss, and limb spasticity. Comprehensive rehabilitation at a referral rehabilitation center improved her condition, and she was able to ambulate with a walker at discharge. She wore a cervical collar for 6 months postoperatively and entered vocational rehabilitation. At 8 months post-surgery, she was walking independently with minor hand issues.

## Discussion

Congenital anomalies of the posterior C1 arch, a subset of cervical vertebral malformations, occur in up to 3% of population [4]. While typically asymptomatic, these anomalies can manifest with various clinical presentations. Pre-existing skeletal dysplasia may exacerbate degeneration by altering biomechanics, as seen in this case, where an asymptomatic congenital anomaly progressed to arthrosis, atlantoaxial instability and symptomatic myelopathy [5, 6]. Despite the significant risks of injuring nearby vital structures, MRI-confirmed myelopathy often necessitates decompressive surgery and segmental stabilization to prevent symptom progression, as damaged upper motor neuron recovery is typically limited [6].

A posterior approach remains the preferred technique for upper cervical decompression and typically necessitates C1-C2 fusion, with various fixation methods available. The freehand technique for C1-C2 screw placement has demonstrated effectiveness even in cases of bony anomalies [7], and C1 screws can be safely placed with a low risk of VA or neurological injury, with or without CT-guided navigation [8]. Unilateral pedicle fixation at the atlantoaxial joint has shown comparable outcomes to bilateral fixation for fractures, with both approaches recommending rigid cervical collar use for at least 3 months postoperatively to aid fusion [9]. In cases involving lower cervical soft-tissue tumors, 3 months of collar use has also proven sufficient for achieving fusion with unilateral pedicle fixation [10]. However, unilateral lateral mass screw fixation has been associated with anterolisthesis of the upper vertebra in approximately half of reported cases [10]. Notably, neither study included decompressive laminectomy, which further destabilizes the segments. In our case, due to dysplastic anatomy and VA injury, salvage contralateral unilateral atlantoaxial pedicle fixation with C1 laminectomy was performed. This required prolonged immobilization with a rigid cervical collar for 6 months, after which radiographic imaging confirmed successful fusion. For cases involving aberrant VA anatomy requiring C1-C2 fusion, unilateral contralateral fusion using a C1 lateral mass screw and a C2 transarticular screw may also provide a viable treatment option [11].

VA injuries occur in ~1.4% of cervical spine surgeries, with higher rates reported in posterior upper cervical procedures (4%–8%), and vary based on skeletal and vascular anatomy [12]. Management aims to control hemorrhage, prevent central nervous system ischemia, and avoid embolism or pseudoaneurysm [12]. Although primary repair is ideal, it is often unfeasible, requiring occlusion via ligation or endovascular methods [12, 13]. In our case, occlusion was chosen as the injured VA was nondominant, with retrograde flow maintained. The patient's partial Wallenberg syndrome and cerebellar ischemic lesions likely resulted from impaired PICA flow perioperatively. Despite these complications, rehabilitation potential remains high, as shown by the patient’s near-complete recovery.

In retrospect, the use of CT-guided navigation or robotic assistance during screw insertion would likely have reduced the risk of VA injury in this case. Unfortunately, such advanced technologies were not available at our institution at the time. Additionally, creating a pilot hole in the C1 lateral mass with a high-speed drill may have prevented drill bit skidding, thereby decreasing the risk of accidental arterial injury. Also, a high-speed drill in continuous rotation is less likely to directly penetrate the arterial wall. Based on the insights gained from this case, our treatment algorithm has evolved to incorporate these preventive measures in similar high-risk surgical scenarios.

This case highlights the challenges of managing congenital cervical anomalies with VA injury during surgery. Prompt salvage fixation, endovascular intervention, and rehabilitation enabled significant recovery. Early recognition of vascular risks and tailored surgical approaches are crucial for minimizing complications and optimizing outcomes in complex cervical spine procedures.

## Data Availability

This manuscript presents a case report and therefore the data is not available publicly or upon request to protect the privacy and identity of the patient.
